# Impact of extrusion processing conditions on lipid peroxidation and storage stability of full-fat flaxseed meal

**DOI:** 10.1186/s12944-015-0076-4

**Published:** 2015-08-19

**Authors:** Muhammad Imran, Faqir Muhammad Anjum, Nazir Ahmad, Muhammad Kamran Khan, Zarina Mushtaq, Muhammad Nadeem, Shahzad Hussain

**Affiliations:** Institute of Home and Food Sciences, Faculty of Science and Technology, Government College University, Faisalabad, 38000 Pakistan; Department of Dairy Technology, University of Veterinary and Animal Sciences, Lahore, Pakistan; Department of Food Science and Nutrition, College of Food and Agricultural Sciences, King Saud University, Riyadh, Saudi Arabia

**Keywords:** Flaxseed meal, Anti-nutritional compounds, Alpha-linolenic acid, Hot extrusion, Lipid peroxidation, Storage stability

## Abstract

**Background:**

The full-fat flaxseed (*Linum usitatissimum* L.) meal has obtained relatively new flourished concept as food or feedstuff for the development of healthier products. It provides favorable balance of polyunsaturated, monounsaturated and saturated fatty acids. However, flaxseed meal may be susceptible to oxidation by exposure to various storage conditions which is extremely undesirable and produces toxic compounds to human health. Another consideration in the application of flaxseed meal relates to the presence of anti-nutritional compounds that need to be minimized using appropriate processing method. The present research work was conducted to evaluate the impact of extrusion processing conditions and storage of full-fat flaxseed meal on functional characteristics such as α-linolenic acid content, lipid peroxidation and sensory attributes.

**Methods:**

The raw flaxseed meal was analyzed for cyanogenic glycosides, tannin and mucilage anti-nutritional compounds. Fatty acids composition was quantified by gas chromatography. The meal was extruded at barrel exit temperature (100–140 °C), screw speed (50–150 rpm), feed rate (30–90 kg/h) and feed moisture (10–30 %) for reduction of anti-nutritional compounds. The raw and extruded meals were stored for a ninety-day period under room conditions (20–25 °C). Lipid peroxidation was analyzed by peroxide, free fatty acids, conjugated dienes, total volatiles and malondialdehyde assay. Color, aroma and overall acceptability attributes were evaluated by sensory multiple comparison tests.

**Results:**

The raw flaxseed meal possessed significant amount of anti-nutritional compounds, lipid and α-linolenic acid contents. The extrusion processing at high barrel exit temperature (140 °C) significantly reduced the cyanogenic compounds (84 %), tannin (73 %) and mucilage (27 %) in the flaxseed meal. The α-linolenic acid content and lipid peroxidation did not significantly change after extrusion processing or during storage at the end of 60 days. Fluctuations in sensory attributes occurred during storage, but at the end of 90 days, only the extruded samples presented negative effect and showed lowest consumer acceptability.

**Conclusions:**

The present study suggested that extrusion of flaxseed meal at optimum conditions and stored for 60 days did not change the stability of full-fat flaxseed meal and can be used as supplement or ingredient for the production of various healthier products.

## Background

Flax (*Linum usitatissimum* L.), an herbaceous plant, produces seeds which are oval and flattened in shape, 4–6 cm long, pale to dark brown and shiny. Flax production in the world was about 2.6 million tonnes during 2012–2013 and represents 1 % of total world oilseeds supply. The most important flaxseed producing countries are Canada, USA, China, India, Pakistan, Africa and Europe continents. Whole flaxseed meal is attractive for high dietary fiber, protein, phytochemicals and lipid composition. Flaxseed meal is used as supplement or ingredient in various food and feed products and is gaining popularity in the breakfast cereals, pet food, animal and poultry feed industry [1]. Most of the known biological activities of flaxseed meal have been assigned to omega fatty acids (55–60 % of lipids profile) present in the flaxseed. Due to unsaturation nature, flaxseed meal is subject to rapid oxidation and has a limited shelf life. Intake of foods supplemented with oxidized lipid constituents can modify DNA, proteins, membrane structure and tumor initiation [2, 3].

Different predictive and indicator methods such as peroxide value (PV), free fatty acids (FFA) and conjugated dienes values have been applied to investigate the oxidation reactions of flaxseed lipids. Lipid oxidation produces a variety of volatile compounds which tend to increase during storage. Malondialdehyde (MDA), potentially mutagenic, condenses with two equivalents of thiobarbituric acid to give a fluorescent red derivative that can be assayed by spectrophotometrically [4, 5].

The presence of cyanogenic glycosides, tannin and mucilage potentially limit the bulk supplementation of flaxseed meal at commercial scale. This situation demands more attention to reduce the toxic effect and improve the nutritional quality of flaxseed through effective and economical thermal processing. The extrusion processing with controlled conditions has been proposed as an effective approach to remove the anti-nutrients in raw materials [6]. To the best of our knowledge, no study has been published on extruded full-fat flaxseed meal production and further evaluation of its stability at different storage intervals. The objective of this research was to produce detoxified flaxseed meal using single-screw extrusion cooking at different operating conditions and investigate the storage stability of extruded flaxseed meal.

## Methods

The flaxseed cv. *Chandni* was procured from Oilseeds Research Institute, Faisalabad, Pakistan. The seeds of the variety were cleaned to remove any debris or field dirt and any other extraneous matter and stored in sealed polyethylene bags at 5 ± 1 °C.

### Chemical composition of raw flaxseed

The alkaline titration method was used for the determination of cyanogenic contents in flaxseed samples [7]. The concentration of tannin in the flaxseed meal samples was measured by Folin–Denis method with minor modifications [8]. Flaxseed mucilage was analyzed according to the method described by Kaewmanee et al. [9]. The oil from flaxseed samples was extracted through solvent extraction technique using soxhlet apparatus (Model: H-2 1045 Extraction Unit, Hoganas, Sweden) according to AACC [10] Method No. 30–25. The fatty acids profile of extracted oil from flaxseed samples was analyzed by the method Ce 1f–96 given in AOCS [11].

### Hot extrusion processing of flaxseed

A single-screw extruder, Extru-tech E325 (Extru-tech, Sabetha, Kansas, USA) was used for the production of detoxified full-fat flaxseed meal. The commercially available single-screw cooker E325 consists of feed delivery system, preconditioning system, extruder barrel, terminal die, knife assemblies and TEFC electric motor as power transmission system (250HP, 1500 RPM and 380 Volt). The extruder barrel assembly consisted of screws (83 mm diameter), barrel (254 mm diameter) and barrel length to diameter ratio of 9:1. The extruder was divided into six zones along the length of the barrel, with zone–1 designated as feed section and zone–6 nearest the die section. The screws and steamlocks configuration were arranged in such a manner to provide a progressively tighter pitch and greater resistance from feed section to die zone. The raw material was propelled from feed section (wide flight tapered screws, zone–1) into the interior of the kneading section (intermediate flight spacing screws, zones-2, 3 and 4) where the material was compressed to increase the degree of fill of the flow channels. In the final cooking section (tight flight screws, zones–5 and 6), temperature and pressure was increased rapidly. The extruder was operated at barrel exit temperature (BET, 100–140 °C), screw speed (SS, 50–150 rpm), feed rate (FR, 30–90 kg/h) and feed moisture (FM, 10–30 %). The moisture content of flaxseed meal was adjusted at 10 %, 20 % and 30 % by injecting distilled water and stored for 12 h at 5 ± 1 °C before extrusion processing. The extruded samples were cooled down to room temperature and divided into 1 kg samples, packed in polypropylene bags and sealed manually. The flours were stored, without light control, at room temperature (20–25 °C), for ninety–days.

### Storage stability of raw and extruded flaxseed meal

Peroxide value of oils extracted from flaxseed meal samples was determined by AOCS [11] Method No. Cd 8–53. Free fatty acid value of sample oils was analyzed by AOCS [11] Method No. Ca 5a–40. The conjugated double bonds were measured using European Communities official methods [12]. Total volatile components were assessed by gas chromatographic analysis of headspace as reported by Przybylski [13]. The malondialdehyde test was used to calculate the lipid peroxidation according to Kirk and Sawyer [14] method with some modifications.

### Sensory evaluation

Fourteen panel judges consisting of experienced and untrained panelists carried out the sensory analysis of fresh and stored flaxseed meal samples. Sensory parameters were evaluated using the multiple comparison tests according to instructions of Morais et al. [15]. Each panelist received a standard sample (flaxseed meal without extrusion heat treatment) identified by the letter S and experimental samples (extruded flaxseed meal samples) assigned with random three–digit code numbers. Each panelist was asked to list their preference on a 9–cm comparison line (1 = dislike extremely to 9 = like extremely) and a score of 5 was considered equal to the standard. The sensory analysis was performed and completed at 0, 30, 60 and 90 days of storage interval for experimental treatments.

### Statistical analysis

The data of anti-nutritional compounds, oil yield and fatty acids composition obtained for each extrusion parameter (barrel exit temperature, screw speed, feed rate and feed moisture) was subjected to statistical analysis to determine the level of significance by using the software package (Statistic 8.1) according to the method described [16]. The average of the three runs was reported as the measured value with standard deviation. The Duncan’s multiple range (DMR) test was used to estimate the level of significance that existed between the mean values. The sample analysis for storage stability were carried out in triplicate and calculated the significant differences among means at a probability level of 5 %.

## Results and discussion

### Chemical characterization of raw flaxseed meal

The raw flaxseed possessed hydrocyanic acid (198.4 ± 0.6 mg/kg), tannin (146.3 ± 0.5 mg/100 g) and mucilage (8.9 ± 0.7 g/100 g), respectively. The concentration of hydrocyanic acid has been the subject of extensive investigation as contributed by Chadha et al. [17], Park et al. [18] and Kobaisy et al. [19] who predicted hydrocyanic acid contents in the range of 120 to 762 mg/kg for flaxseed cultivars. The considerable variations in reported production of cyanogenic compounds may be attributed to a result of adjustment in flaxseed nutritive composition, environmental conditions and plant developmental stage [20]. The description focusing on presence of tannin in whole flaxseed or flaxseed meal has been examined earlier in limited laboratory studies. Amarowicz et al. [21] have found tannin content in defatted flaxseed meal ranged from 125 to 137 mg/100 g. Wanasundara and Shahidi [22] also reported condensed tannin in the range of 130 to 136 mg/100 g flaxseed meal. The mucilage yield from seven different cultivars of whole flaxseed using specific extraction conditions ranged from 1.8 % to 3.6 % of fresh seed weight [9]. These values are lower than those reported in present study. The yield of mucilage largely depends upon the extraction method, time and temperature [23]. The average oil contents in raw flaxseed were found to be 32.2 ± 0.4 g/100 g. The fat content recorded in the present study is quite in agreement with those values reported by Khan et al. [24] who found oil percentage 36.6 g/100 g in flaxseed. The oleic, linolenic and α-linolenic have been found predominant fatty acids in flaxseed meal. The oleic, linoleic and α-linolenic acids found in tested raw flaxseed samples were 18.6 ± 0.3 %, 11.8 ± 0.4 % and 50.2 ± 0.7 %, respectively. The distribution pattern of oleic and linoleic fatty acids were found 14.8–22 % and 16.1–18.2 %, respectively [25]. The α-linolenic as major fatty acid was present in flaxseed. The α-linolenic content found in raw flaxseed in the present study is supported by results of Nykter et al. [26] who found α-linolenic acid in the range of 52.8 % to 60.4 %.

### Extrusion processing and anti-nutritional compounds

In the present study, the extrusion cooking significantly decreased the anti-nutritional compounds in raw flaxseed meal (Table 1). The BET was shown to impart a strong effect on reduction of anti-nutritional compounds in flaxseed meal. The reduction rate of anti-nutritional compounds in flaxseed was slow at low temperature range during the initial point (100 °C) of extrusion. However, it appears that the increase in temperature (140 °C) may improve the reduction of anti-nutritional compounds. After the heat treatment at 140 °C, the extruded flaxseed meal contained significantly (p ≤ 0.05) low level of cyanogenic glycosides (31.5 ± 0.6 mg/kg), tannin (39.2 ± 0.5 mg/100 g) and mucilage (6.5 ± 0.2 g/100 g) compounds. The action of SS and FR had minimal effect on the anti-nutritional compounds reduction. The results show that SS and FR represent a very active source of anti-nutritional compounds reduction on low level, but at high SS and FR, it leads towards a negative decrease in reduction action. Similar results were observed with FM contents. Twin-screw extrusion with optimum moisture content (16.6 %); barrel exit temperature (156 °C), screw speed (219 rpm) and feeding speed (76.1 kg/h) increased the nutritional quality of flaxseed through removal of hydrocyanic acid content [27]. The optimized results for barrel temperature of 146.0 °C and feed rate of 32.7 kg/h exhibited maximum rate of HCN removal (93.2 %) during the twin-screw extrusion detoxification technique on flaxseed via stepwise non-linear response surface methodology [28]. Mukhopadhyay et al. [29] has predicted maximum reduction 61.2 % of tannin in linseed meal at optimum values of barrel exit temperature 82.5 °C and screw speed 90 rpm. The maximum removal rate of mucilage (60.3 %) from flaxseed meal was achieved at die temperatures (80–160 °C), screw speeds (300–900 rpm) and initial moisture contents (18.8 %–35.1 %) of co-rotating twin-screw extruder [30]. The extrusion cooking improved the degradation of flaxseed mucilage and negatively influenced the flow behavior index of compact fiber structure with the addition of initial moisture content, high temperature and decreased screw speed [31].

**Table 1:** Chemical composition of extruded full-fat flaxseed meals at different extrusion processing conditions

Extrusion conditions		Chemical composition						
		Cyanogenic compounds (mg/kg)	Tannin (mg/100 g)	Mucilage (g/100 g)	Oil yield (g/100 g)	Fatty acids		
						Oleic (% of TFA)	Linoleic (% of TFA)	α-Linolenic (% of TFA)
Barrel exit temperature (°C)	100	52.3 ± 0.4^e^	59.3 ± 0.6^e^	7.6 ± 0.2^c^	31.6 ± 0.1^b^	18.6 ± 0.1^a^	11.4 ± 0.2^a^	47.7 ± 0.4^a^
	120	45.1 ± 0.5^f^	51.4 ± 0.6^h^	7.1 ± 0.2^f^	30.2 ± 0.2^c^	18.8 ± 0.2^a^	11.3 ± 0.1^a^	47.1 ± 0.5^a^
	140	31.5 ± 0.6^h^	39.2 ± 0.5^j^	6.5 ± 0.2^h^	29.8 ± 0.2^c^	19.1 ± 0.1^a^	11.1 ± 0.2^a^	46.6 ± 0.6^a^
Screw speed (rpm)	50	70.5 ± 0.5^a^	74.8 ± 0.4^a^	7.3 ± 0.3^e^	31.3 ± 0.2^b^	19.0 ± 0.2^a^	11.4 ± 0.2^a^	49.0 ± 0.6^a^
	100	52.9 ± 0.4^e^	53.1 ± 0.6^g^	7.5 ± 0.3^c^	29.0 ± 0.1^d^	18.7 ± 0.2 ^a^	11.5 ± 0.2^a^	48.3 ± 0.5^a^
	150	61.8 ± 0.5^c^	64.9 ± 0.7^c^	7.9 ± 0.2^a^	31.5 ± 0.1^b^	18.4 ± 0.1^a^	11.7 ± 0.1^a^	49.1 ± 0.5^a^
Feed rate (kg/h)	30	59.0 ± 0.5^d^	61.9 ± 0.7^d^	7.4 ± 0.1^d^	31.4 ± 0.2^b^	18.9 ± 0.1^a^	11.4 ± 0.2^a^	49.5 ± 0.6^a^
	60	46.3 ± 0.6^f^	57.1 ± 0.4^f^	6.9 ± 0.3^g^	31.3 ± 0.2^b^	18.7 ± 0.2^a^	11.5 ± 0.3^a^	48.4 ± 0.5^a^
	90	66.7 ± 0.3^b^	72.7 ± 0.8^b^	7.7 ± 0.2^b^	29.9 ± 0.1^c^	18.6 ± 0.2^a^	11.6 ± 0.2^a^	49.2 ± 0.6^a^
Feed moisture (%)	10	26.4 ± 0.3^i^	35.9 ± 0.4^k^	7.0 ± 0.2^g^	31.3 ± 0.2^b^	18.7 ± 0.2^a^	11.4 ± 0.1^a^	48.0 ± 0.6^a^
	20	43.7 ± 0.4^g^	45.9 ± 0.5^i^	7.4 ± 0.1^d^	32.0 ± 0.1^a^	18.5 ± 0.1^a^	11.7 ± 0.1^a^	48.3 ± 0.5^a^
	30	62.4 ± 0.5^c^	58.1 ± 0.6^f^	7.9 ± 0.2^a^	32.1 ± 0.1^a^	18.5 ± 0.1^a^	11.8 ± 0.2^a^	48.6 ± 0.5^a^

Values represent the mean ± standard deviation; n = 3

^a–k^Means in a column with different superscripts were significantly different (p < 0.05)

TFA = Total fatty acids

The effect of single condition of extrusion was determined by setting the all other three conditions at mean values, respectively

### Extrusion processing, oil yield and fatty acids composition

The extraction yield values of oil from flaxseed samples differed after the extrusion cooking (Table 1). The oil yield decreased exponentially with increasing BET. The minimum value (29.8 ± 0.2 g/100 g) of oil contents was found in extruded flaxseed meal samples when BET was set at 140 °C. The speed of screws rotation was found inversely proportional to the oil yield. The higher the rotation of screws, the higher was the oil contents (31.5 ± 0.1 g/100 g) in extruded flaxseed meal samples. This difference might be due to retention time which decreased by increasing the rotation of screws. Besides, when FR was increased, the oil contents also decreased in tested samples. This is because at high FR, more feed material was inside the barrel cavity which resulted more percolation of oil. While on increasing FM contents, the oil yield remained constant when compared to initial material. The oil contents have been found to decrease from 42 % to 30 % after extrusion processing of seed flakes [32]. This effect of processing conditions can be attributed to disruption of cell walls which increased the migration of oil outside from raw material and produces meal samples with less oil contents [33].

The results demonstrated that the contents of oleic, linoleic and α-linolenic acid were stable (p ≥ 0.05) after the application of extrusion processing. At the same time, the relative content of oleic acid in extruded flaxseed samples increased when temperature of barrel was increased during processing (Table 1). The barrel exit temperature was seen to be as major factor affecting the essential fatty acids composition of flaxseed meal samples. The α-linolenic acid contents decreased non-significantly on increasing barrel exit temperature and keeping the other three extrusion conditions i.e., SS, FR and FM constant at medium level. This behavior can be attributed to transition of α-linolenic acid content in more saturated state characterized with smaller number of double bonds [34]. The α-linolenic acid content in extruded flax samples decreased with decrease in rotation of screws. The application of high screw speed produced the extruded flax meal samples with high α-linolenic acid content which might be due to short stay phenomena of feed material in the extruder cavity. It can be observed from Table 1 that the α-linolenic acid was non-significantly reduced in flaxseed meal samples at mean level of FR and FM conditions in extruder barrel cavity. However, increase in α-linolenic acid retention was observed when feed rate was exceeded from mean point. Wicklund and Magnus [35] found a non-significant effect of extrusion temperature range from 120 °C to 150 °C on percentage distribution of α-linolenic acid during sifted oat flour. The extrusion of raw and pre-conditioned linseed at a temperature of 120 °C showed no significant reduction of α-linolenic fatty acids in feed material [36].

### Extrusion processing, storage and lipid peroxidation

**Fig 1:**
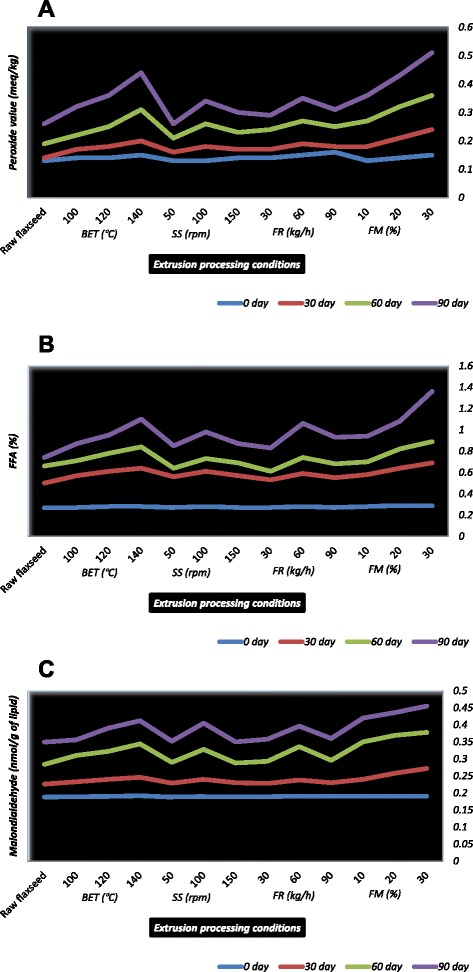
Effect of extrusion processing conditions and storage period on lipid oxidation of full-fat flaxseed meal (**a**: Peroxide value, **b**: Free fatty acids value and **c**: Malondialdehyde value)

The effects of various extrusion conditions and storage on lipid peroxidation indicators of the flaxseed meal are shown in Fig. 1. The raw flaxseed meal samples showed non-significant changes in PV, FFA, conjugated dienes, total volatiles, malondialdehyde, linoleic and α-linolenic acid values throughout the 90 days storage period. The different extruded flaxseed meal samples started with similar PV (between 0.13 and 0.16 meq/kg) and the slope of the initial change with storage time was also similar (Fig. 1a). The PV of samples extruded at BET (140 °C), SS (100 rpm), FR (60 kg/h) and FM (30 %) reached their peaks after 90 days. Peroxide levels of the flaxseed oil cake were below the threshold limits after 6 months storage at 20 °C [37]. Both Linott and the mixed variety flaxseed were stable over 128 day of storage at 23 ± 2 °C as measured by PV [38]. The composition of flaxseed changed only slightly during storage over 6 months [39]. The PV of oil extracted from flaxseed meal, an empirical measure of oxidation products, is approximately 2 [40]. The peroxides are considered as early oxidation products with relatively short induction periods during which they form, accumulate and dissipate [41]. It seems true that the extruded flaxseed meal samples stored for 90 days were relatively stable and never exceeded the limit of 10 (meq/kg) PV considered as a threshold limit [14].

The samples of extruded flaxseed meal were considered to be of good quality as indicated by low levels of FFA initially. No significant (*p* ≤ 0.05) changes in FFA were found at the end of 30 days for extruded samples treated at low or extreme BET, SS, FR and FM conditions. FFA contents increased when operating temperature, moisture level of initial feed material, and duration of the storage increased (Fig. 1b). The non-vacuum oil cake packed samples stored at 4 °C showed the increase in FFA value from 0.9 to 2.4 % after 6 months [37]. It has been generally observed that the FFA value of samples increased with storage time and increasing temperature [42]. The FFA values above 2 % are considered as onset of rancidity [14]. The oxidation and high values of FFA indicate that the toxic compounds have been produced in raw or processed material and the oxidized products may be associated with the rancid taste and development of cancer and atherosclerosis in biological system [40]. The elevated amount of FFA nearly 10 % was found in the milled samples of flaxseed stored for 11 months which was likely due to the presence of sufficient moisture to allow lipolytic activity to occur during storage [43]. A similar range of conjugated dienes was observed among the extruded flaxseed meal samples indicating that the samples have similar initial quality. The non-significantly increasing order for conjugated dienes of raw flaxseed meal during storage intervals was found 0.28 < 0.30 < 0.32 < 0.34 (0, 30, 60 and 90 days). The extruded flaxseed meal samples followed the order for conjugated dienes as 0.20 < 0.24 < 0.27 (BET, 100, 120, 140 °C), 0.23 < 0.25 < 0.26 (SS, 50, 150, 100 rpm), 0.21 < 0.25 < 0.26 (FR, 30, 90, 60 kg/h), and 0.26 < 0.27 < 0.28 for FM (10, 20, 30 %), respectively at the end of the study trial. Conjugated dienes, primary oxidation products, were not detected in the stored milled flaxseed samples which suggest good oxidative stability of milled flaxseeds [43].

The levels of total volatile compounds obtained from 30 to 90 days of extruded flaxseed samples were higher when compared to initial readings. The maximum values for samples extruded at 140 °C BET (2510 ppb), 100 rpm SS (2490 ppb), 60 kg/h FR (2440 ppb) and 30 % FM (2480 ppb) conditions were observed after the 90 days of storage. The raw flaxseed meal showed a similar behavior for total volatile compounds production; however, produced relatively less amount of compounds (2300 ppb) after the 90 days when compared to extruded samples. Total volatiles were observed to increase with storage in the mixed variety sample of flaxseed but showed minimal change in the Linott sample of flaxseed variety [38]. Fig. 1c shows how the different extrusion processing conditions and storage period influenced the MDA values. The peak in MDA value was reached after 90 days with final values of 0.41 nmol/g of lipid (BET, 140 °C), 0.40 nmol/g of lipid (SS, 100 rpm), 0.4 nmol/g of lipid (FR, 60 kg/h), and 0.5 nmol/g of lipid (FM, 30 %). All MDA values behaved similarly (rising trend). However, the oil with the highest FM contents experienced the most drastic changes in MDA value. The MDA value of the raw flaxseed meal samples stayed on a lower level (about 0.3 nmol/g of lipid) at the expiry of study period. These results demonstrated that flaxseed meal is stable in heat treatment under the conditions initially used. The polyunsaturated fats containing three or more double bonds are more sensitive for higher MDA values, but is not so sensitive for the oxidation products of oleic and linoleic acid. The greater the MDA value, the more the samples contains oxidation products [44]. The final concentration of malonaldehyde in the partially defatted flaxseed meal sample stored for fourteen days was found small [45]. The MDA concentration in the heat treated brown flaxseed whole meal ranged from 0.005 to 0.405 nmol of MDA equivalents per gram of lipid, and from 0.006 to 0.265 nmol of MDA equivalents per gram of lipid in the raw seed meal [15]. Nonvacuum-packed flaxseed oil cake samples stored at 20 °C showed the highest increase in TBA values from 0.012 to 0.021 over 6 months of storage [37]. The whole flaxseed remains stable in terms of lipid oxidation for many years; however, cold-pressing and high moisture conditions during storage can trigger enzymatic-promoted oxidation [46].

In addition to lipid oxidation indicators, the α-linolenic acid contents are also considered as an indicator of flaxseed meal suitability for incorporation in healthy foods for discerning consumers. At day zero, the raw flaxseed meal presented the highest values of α-linolenic acid. These values tended to slightly decrease during the storage period (48.3 ± 0.4 % and 46.2 ± 0.3 %, respectively at 60 and 90 days). It seems that raw flaxseed meal was less influenced by storage time, showing a less marked decreasing trend over time. During storage, a marked decrease in α-linolenic acid content was observed for extruded samples. The flaxseed meal samples treated with low BET, SS, FR and FM conditions had a lower loss (13 %) of α-linolenic acid during 90 days storage. On the contrary, the flaxseed meal samples exposed to high BET and FM conditions at optimum level of SS and FR showed loss (22 %) in α-linolenic acid content at the end of 90 days study period. After heating to 178 °C for 1.5 h, α-linolenic acid decreased from 55.1 % to 51.3 % in ground flaxseed, and to 51.7 % in lipid extracts, but it remained unchanged in the whole flaxseed [47]. Most of the lipid oxidation detected in milled flaxseed samples occurred on the surface that was exposed to the air during storage for 48 days at 50 °C [48]. The α-linolenic acid content of brown flaxseed whole flour did not significantly change after heat treatment in oven at 150 °C for 15 minutes or during storage for a thirty-day period under similar conditions to those used commercially [15]. Likewise, Malcolmson et al. [38] also reported that meals from two flaxseed varieties stored for 128 days at room temperature and protected from light presented no changes in their α-linolenic acid content. The existence of endogenous phenolic antioxidants in the milled flaxseed matrix may account for the protection of polyunsaturated fatty acids and stability against oxidation. The presence of such phenolic compounds results in decreasing rate of off-flavors development [26]. The present study shows that storage and heat partially decrease the amount of α-linolenic acid content. Therefore this must be taken into consideration when selecting the operating temperature and storage conditions.

### Extrusion processing, storage and sensory attributes

**Fig 2:**
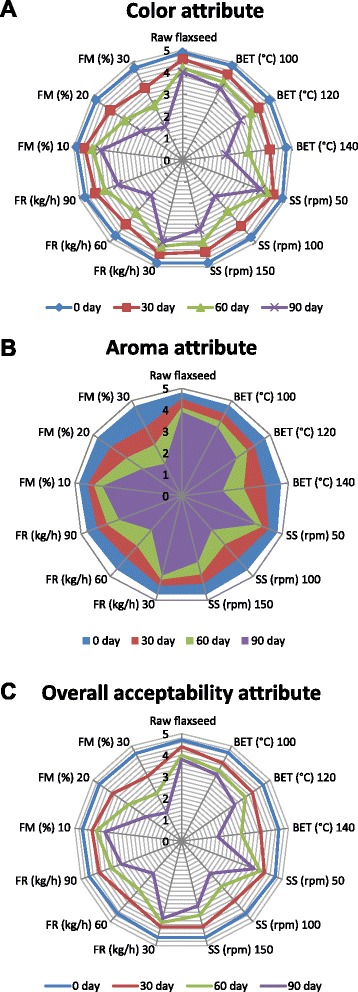
Effect of extrusion processing conditions and storage period on sensory attributes of full-fat flaxseed meal (**a**: Color, **b**: Aroma and **c**: Overall acceptability)

The results of sensory characteristics point out the differences between samples acceptability and also indicate the changes in evaluated sensory properties after 90 days of storage time. The results from Fig. 2 indicated that the extrusion processing and storage negatively affected the preservation of color and aroma of the flaxseed meal samples. The flaxseed meal samples obtained from raw seeds showed color (Fig. 2a) and aroma (Fig. 2b) stability higher than the extruded meal samples. The sensory panel could not almost detect any differences in the sensory properties of fresh or stored raw flaxseed meal samples. Average sensory scores of assigned to color, aroma and overall acceptability (Fig. 2c) attributes varied in a quite narrow range for all extruded flaxseed meal samples at the initial days of storage., regardless of applied extrusion processing conditions. The lowest evaluation scores were recorded for samples extruded at high BET and FM conditions after 90 days. Morais et al. [15] found significant difference for tested sensory parameters among the brown flaxseed whole flour samples treated in oven at 150 °C for 15 minutes and stored for thirty days, and scores remained between equal and slightly below the standard. However; no effect of time of storage on aroma and color attributes was found for raw meal in the same study. The assessors were not able to record a difference in odor characteristics among the fresh and stored flaxseed samples [38].

## Conclusions

The results of present study conclude that raw flaxseed meal possessed the highest oxidative stability. The anti-nutritional compounds were found quite low in extruded flaxseed samples with non-significant changes in essential fatty acids. The oxidation level of flaxseed meal samples increased non-significantly after extrusion processing and during the storage period. The acceptability of extruded samples decreased with increasing storage time. Depending upon these sensory results it can be recommended that flaxseed meal processed at high barrel temperature and feed moisture conditions will be suitable to use for production of various healthier products during 60 days. Additional studies should be undertaken to determine the maximal shelf life of products supplemented with flaxseed meal processed at different extrusion conditions.
